# Influence on fluid dynamics of coronary artery outlet angle variation in artificial aortic root prosthesis

**DOI:** 10.1186/1475-925X-7-9

**Published:** 2008-02-28

**Authors:** Janko F Verhey, Christoph Bara

**Affiliations:** 1 MVIP ImagingProducts GmbH, Nörten-Hardenberg, Germany; 2 Department of Medical Informatics, University Hospital, Göttingen, Germany; 3 Department of Thoracic and Cardiovascular Surgery, Hannover Medical School, Hannover, Germany

## Abstract

**Background:**

Because of higher life expectancy, the number of elderly patients today with degenerative aortic diseases is on the increase. Often artificial aortic roots are needed to replace the native tissue. This surgical procedure requires re-implantation of the previous separated coronary arteries into the wall of the prosthesis. Regardless of the prosthesis type, changes in the reinsertion technique, e.g., the variation of the outlet angle of the coronary arteries, could influence the coronary blood flow. Whether the prosthesis type or the outlet angle variation significantly improves the blood circulation and lowers the risk of coronary insufficiency is still an open question. The numerical calculations presented can help to clear up these disputable questions.

**Methods:**

Two simplified base geometries are used for simulating the blood flow in order to determine velocity and pressure distributions. One model uses a straight cylindrical tube to approximate the aortic root geometry; the other uses a sinus design with pseudosinuses of Valsalva. The coronary outlet angle of the right coronary artery was discretely modified in both models in the range from 60° to 120°. The pressure and velocity distributions of both models are compared in the ascending aorta as well as in the right and the left coronary artery.

**Results:**

The potentially allowed and anatomic limited variation of the outlet angle influences the pressure only a little bit and shows a very slight relative maximum between 70° and 90°. The sinus design and variations of the outlet angle of the coronary arteries were able to minimally optimize the perfusion pressure and the velocities in the coronary circulation, although the degree of such changes is rather low and would probably not achieve any clinical influence.

**Conclusion:**

Our results show that surgeons should feel relatively free to vary the outlet angle within the anatomic structural conditions when employing the technique of coronary reinsertion.

## Background

The first surgical technique of valve-sparing aortic root reconstruction was described by [1,2]. Since then aortic surgery has developed rapidly, for the most part without changing the bases of the technique [3]. Valve-sparing aortic root replacement has provided very good results and has gained increasing acceptance over time [4,5]. The main limitation of this operation, however, is the need for an intact structure of the aortic valve to successfully complete the reconstruction. The gold standard for root replacement in all other cases is still the composite replacement by a Dacron tube carrying a mechanical or biological valve [6,7].

The excellent results of aortic surgery as well as the increasing amount of degenerative aortic disease due to the rising number of elderly patients in the last years has produced continuing interest in this procedure. Some reports point to the importance of sinus wall compliance [8], the well-known natural "Windkessel" mechanism [9] that causes a continuous aortic flow despite pulsatile cardiac output, and to the crucial role of the Valsalva sinuses [10]. This effect is lost in a stiff Dacron tube without any kind of bulbous. The new type of artificial aortic root prosthesis with pseudosinuses of Valsalva was developed in order to improve blood flow characteristics,. Some authors have clinically tested and compared both types of root prosthesis with and without pseudosinuses [11-13]. There have also been some examinations of the valve function in both types of prosthesis [13-15], but only little data are available regarding the coronary flow characteristics of pressure and velocity [16]. Our study examines the influence of different outlets of the coronaries on coronary blood pressure and velocity in the two different types of the aortic root prosthesis – the more recent model with pseudosinuses of Valsalva and the older design with straight cylindrical tube. Against the background that all of these procedures require the coronary arteries to be re-implanted into the artificial tube, we simulated various angles of reinsertion and examined the consequences for coronary pressure and velocity.

Computational fluid dynamics (CFD) as a computational technology provides detailed performance assessment for design as well as helping to reduce the need for costly experimentation. CFD also enables sophisticated analysis for predicting fluid flow behaviour as well as heat transfer, mass transfer, phase change, chemical reaction, mechanical movement, and the stress or deformation of related structures. CFD allows the user to build a computational model that represents a system or device (e.g., the aortic root) without carrying out costly experiments. Then fluid flow physics [17,18] is applied to the device, and the software makes predictions for fluid dynamics. In practice, the cardiovascular simulation is a coupled problem [19], which makes it difficult to calculate cardiovascular simulations using CFD. Assumptions usually simplify the blood behaviour to be (1) inhomogeneous, (2) anisotropic, (3) non-Newtonian fluid and (4) having rigid boundaries of flow (the arteries, veins, heart, etc.). As a consequence, CFD analysis is basically used to provide an efficient method of carrying out sensitivity studies on key design parameters for selected parts of the heart. In the past, the native as well as artificial geometries of ventricles [20-22], valves and leaflets [23-27], vessels [28], etc., were investigated and modelled. A good recent overview is given by Yoganathan et al. [29].

Such analyses are carried out to identify the most significant parameters for device design. Flow modelling provides engineers with several benefits: (1) the ability to accurately determine the performance of design concepts, (2) a reduction in costs, and (3) the avoidance of physical testing and the building of prototypes.

In this article we focus on the insertion of the coronary arteries into different types of aortic root prosthesis. To the authors' knowledge very few studies even mention the aortic root and ascending aorta region or include them in their models [30-37]. Yet, in 1997, Makhijani et al. [33] emphazised that the computer model has great potential for becoming a powerful design tool for bioprosthetic aortic valves. To date this remains an open challenge.

## Materials and methods

**Figure 1:**
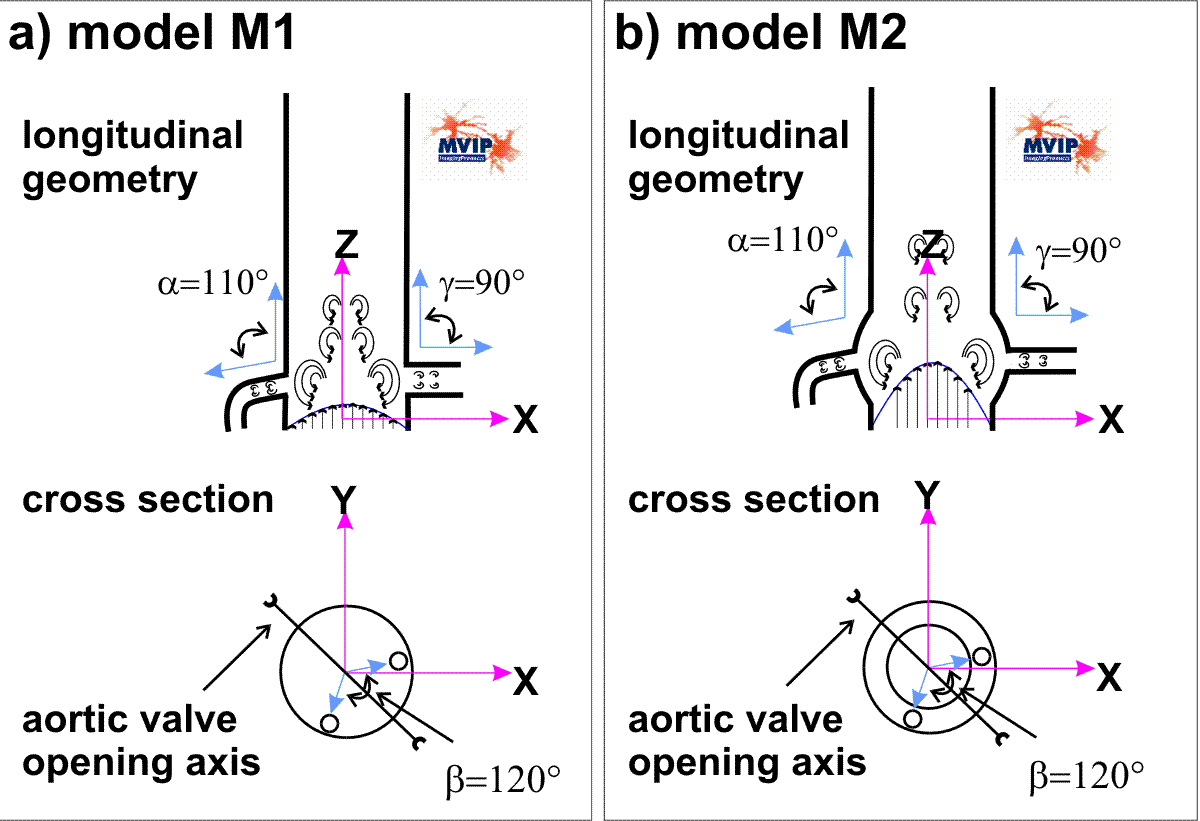
Prosthesis model geometries. (a) Shows the model M1 without "Windkessel" sinus design. (b) Shows the corresponding model M2 with "Windkessel" sinus design. Both geometries are shown in a longitudinal axis (upper part) and in a cross-sectional view (lower part).

The simplified base geometry of the two prostheses described in this article are generated from real anatomical geometries as used in clinical practice. The settings of the two base models are described in Figure 1. One of the base models is created without sinus design (model M1), whereas the other model has a sinus design (model M2, Valsalva sinus or „Windkessel“). All values are set according to average anatomical findings and could be basically modified in the model.

**Figure 2:**
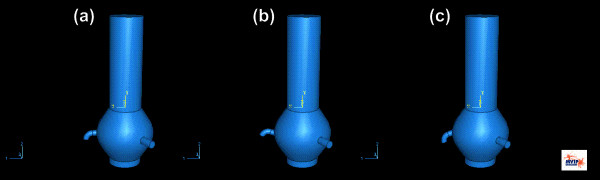
Variation of the right coronary outlet angle (a) 90°, (b) 70° and (c) 110°.

Both models have in common the diameters of the inlet (the aortic valve) and the outlets (the ascending aorta, the left and the right coronary arteries). At the base of the aortic valve a diameter of 25 mm is set. The diameter of the left coronary artery is set to 4 mm and to 3 mm for the right coronary artery, respectively. The ascending part of the aorta has a diameter of 25 mm. The coronary arteries are placed at a distance of 15 mm to the aortic valve. In the model the left artery is straight, has a length of 10 mm, and the coronary outlet angle *γ *is fixed to 90°. The right artery has a curvature of 90° at a length of 3 mm. The coronary outlet-angle *α *of the right coronary artery was discretely modified in the range from 60° to 120° (Figure 2). The angle between the two coronary arteries is set to 120° (Figure 1(a) and 1(b) lower parts).

For model M2 the sinus design is approximated by a toroidal shape with an outer diameter of 5 mm and a length in the longitudinal z-axis of 10 mm, which is equivalent to the length across the axis of the aorta.

In order to have comparable models, the overall lengths of both models are the same. No other geometrical parameter was modified in this study.

The model idealises the fluid dynamic assuming a laminar flow for the velocity profile. The parameters, especially the volume flow l_V_, follow the law of Hagen-Poiseuille:

$$l_{V}=\frac{\pi •r_{AorticValve}^{4}}{8•\eta •l}\Delta p$$

The model deals with a blood viscosity *η *= 3.5·10^-3 ^Pa·s. Both parameter sets for geometry and fluid dynamics idealise the real anatomy and physiology. Some assumptions were made: (1) The tissue of the vessel walls is assumed to be rigid. (2) Blood is treated as homogeneous, isotropic, non-compressible Newtonian fluid with a constant density of 1060 kg/m^3^. (3) The flow distribution at the inlet of the aortic valve region is idealised to a laminar flow. Turbulent components are calculated inside the model. (4) The volume flow rates are set according to the values given in Table 1. An average continuous value (83.33·ml/s) corresponding to physiological findings for the volume flow rate is assumed for the inlet at the aortic valve. (5) During the heart cycle the pressure in the ascending aorta physiologically varies between ~75 mmHg (~9999 Pa) and ~130 mmHg (~17332 Pa). For our calculations we assumed an average pressure of 100 mmHg (= 13332 Pa).

**Table 1:** Boundary conditions for the volume flow rate

Boundary	Type	Volume flow rate [ml/s]
Aortic valve (inlet)	inlet	83.33
Right artery	outlet	1.61
Left artery	outlet	2.55
Asc. aorta (outlet)	outlet	79.17

The base geometries for both models (Figure 1) were generated with the standard finite element program ABAQUS 6.5.1. The CFD calculations were carried out with Fluent Flow Wizard 1.0.8.

## Results

**Figure 3:**
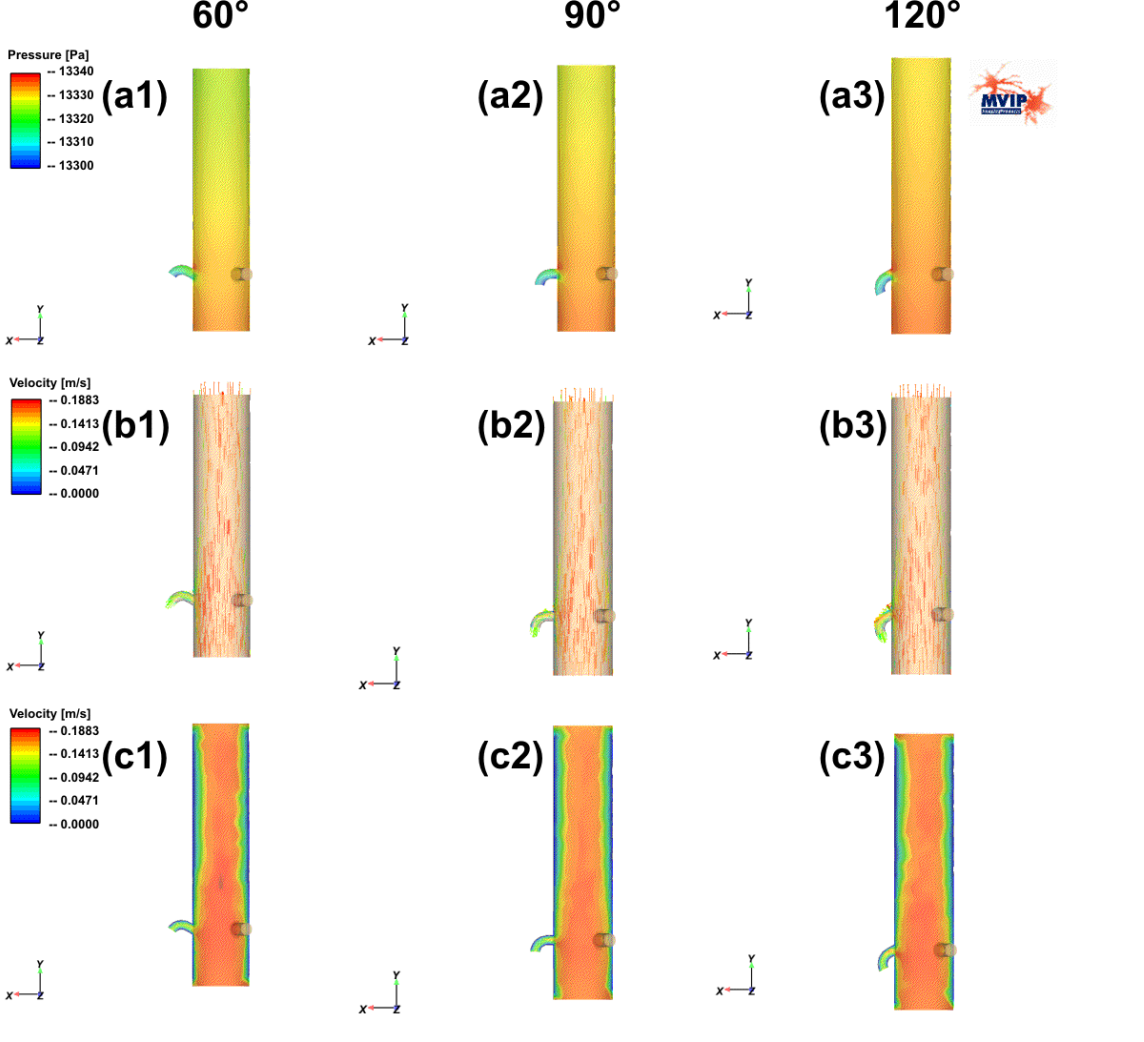
The upper line (a1–a3) shows the pressure distributions for the model M1 for various outlet angles (60°, 90° and 120°) of the right coronary artery. The right coronary artery is on the left side in each image. The middle and the lower rows (b1–c3) show the velocity distributions for the same outlet angle variations in two different visualizations. (b1–b3) Visualize the distributions using colour coded arrows, whereas (c1–c3) do the same with colour-coded areas.

**Figure 4:**
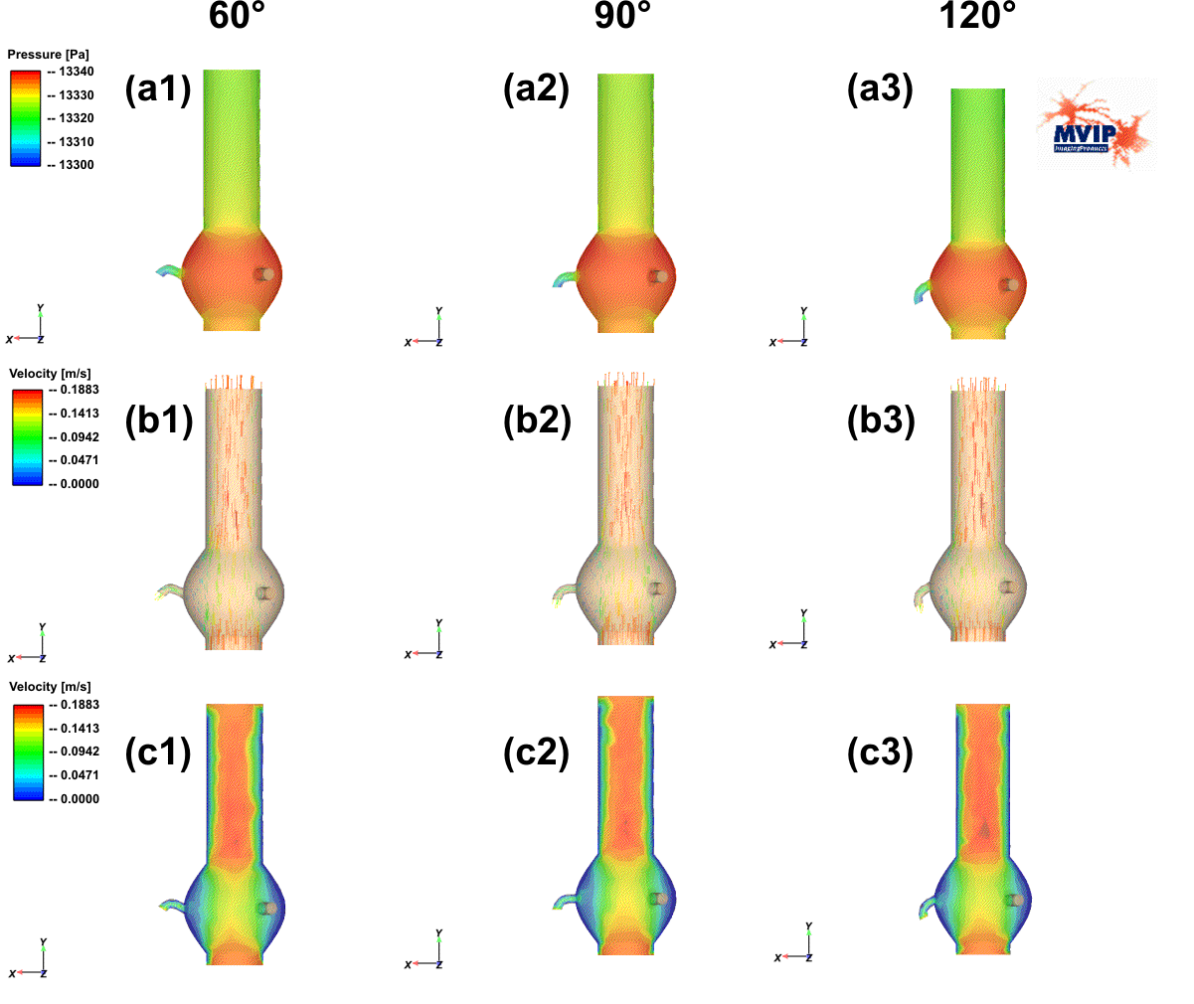
The upper line (a1–a3) shows the pressure distributions for the model M2 with "Windkessel" sinus design for different outlet angles (60°, 90° and 120°) of the right coronary artery. The right coronary artery is on the left side in each image. The middle and the lower rows (b1–c3) show the velocity distributions for the same outlet angle variations in two different visualizations. (b1 – b3) Visualize the distributions using colour-coded arrows, whereas (c1–c3) do the same with colour-coded areas.

**Figure 5:**
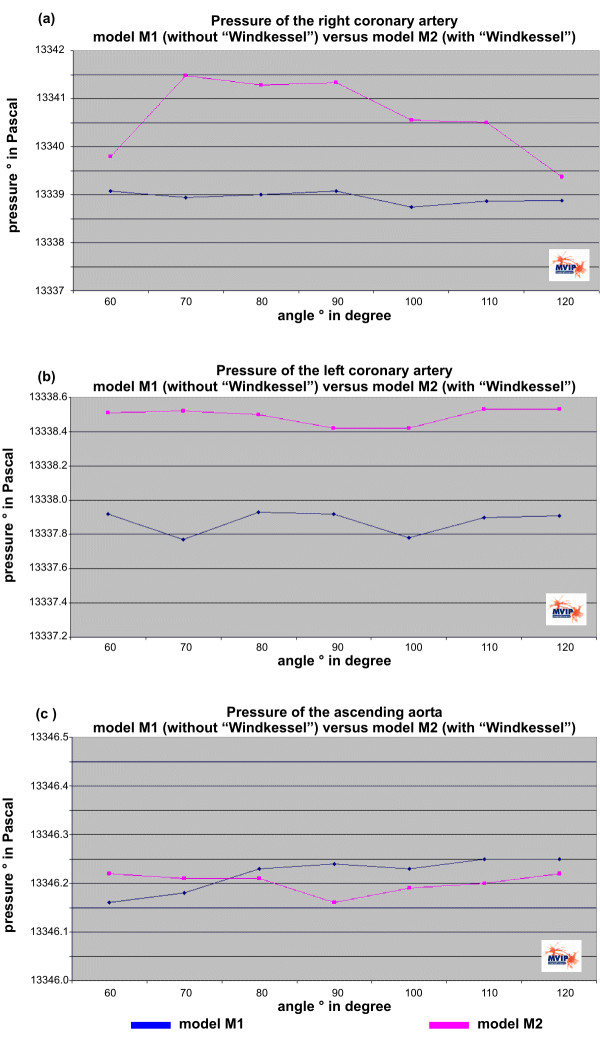
Variation of the outlet angle of the right coronary artery and its effect on the pressure for both models. (a) Shows the perfusion pressure for the right coronary artery. (b) and (c) show similar curves for the left coronary artery and for the ascending aorta. Values for the model M1 are coloured blue, for the model M2 with "Windkessel" sinus design geometry are coloured pink, respectively.

The simulation results for pressure and velocity distributions are shown in Figure 3 and Figure 4. Transversal profiles are presented in both figures. Detailed quantified results for the pressures observed in the three outlets (right and left coronary artery as well as the ascending aorta) are given in the Figure 5.

In all images of Figure 3 and Figure 4 the assumed idealized laminar flow at the aortic valve region is visible because of the homogeneous colour distribution. Turbulent components decrease in strength above the coronary artery region for both models. At the center of the aortic duct the velocity is significantly higher than at the borders of the tube in both models. The region of special interest surrounds the coronary arteries, where the behaviour of pressure and velocity distributions differs between model M1 and M2.

The decrease of pressure in model M1 is continuous and depends on the length of the tube. In each model the influence on the pressure caused by the coronary arteries seems to be negligible (Figure 3 a1–a3). A similar effect is found in the velocity distributions of the model M1: Generally, the turbulence increases in flow direction but the overall velocity distribution profile doesn't change a lot (Figure 3 b1–c3). A slight change in the overall pressure distribution is observed in the variation of the outlet angle of the right coronary artery. The pressure slightly increases in the ascending tube (Figure 3 a1–a3 and Figure 5).

Both the pressure and the velocity distribution differ a little bit at the coronary artery region in the model M2 with sinus design (e.g., Figure 3 a1 versus Figure 4 a1 for the pressure, and Figure 3 c1 versus Figure 4 c1 for the velocity distribution).

Above the coronary artery region the relative distribution changes slightly from model to model. Whereas the pressure in model M1 decreases continuously by the length of the tube (Figure 3 a1–a3), Figure 3 c3 clearly shows a discontinuous change at the end of the "Windkessel" sinus region. The overall pressure decreases in model M2. For all calculations we subtracted the average blood pressure of approx. 100 mmHg (= 13332 Pa) and calculated a difference-pressure, This leads to an average pressure of 4.7 Pa at the outlet of the ascending aorta for the model M2. This is only slightly lower than in model M1, where the difference pressure is 8.5 Pa.

Quantified results are compared for both models in Figure 5. Figure 5a shows the perfusion pressure for the right coronary artery, where it is visible that the difference pressure in model M2 with the „Windkessel“ sinus design is higher than in the model M1. The difference pressure is around 6.5 Pa for all angles for model M1. The „Windkessel“ sinus design causes an increase up to 9.5 Pa with a maximum between 70° and 90°. For the left coronary artery and the ascending aorta the pressure values in Figure 5b and Figure 5c do not show any significant change with the variation of the right coronary artery outlet angle.

## Discussion

Optimizing implanted aortic root prostheses is still a challenge because of their inferiority to the native aorta. One of the proposed solutions to improve the characteristics of the prosthesis is a model using pseudosinuses of Valsalva ("Windkessel") as opposed to a straight cylindrical tube.

Our pressure and velocity distributions show that the coronary blood flow improvement caused by the sinus design seems to be only insignificantly superior to the straight tube model. To the authors' knowledge the only previous investigation of the coronary flow after aortic root replacement was performed by De Paulis et al. [16], who examined patients with a standard cylindrical Dacron conduit, with the sinus-conduit and after aortic valve as well as ascending aortic replacement one year after surgery. They didn't find any influence of pseudosinuses of Valsalva on the coronary flow reserve corresponding to our findings. On the other hand, they did find a greater diastolic component of the flow in group of patients with sinus-design conduit and suggested that the coronary flow pattern may be affected by the presence of sinuses. Our simulations could not confirm these suggestions and thus provide a theoretical backup. The results, however, must be controlled in further clinical investigation by comparative estimation of the coronary flow characterized by pressure and velocity distributions in patients with different types of the aortic prosthesis.

An improvement in coronary flow should be emphasized first of all in order to minimize the future aortic-surgery-related risk of coronary incidents. Any intervention should lead to optimal pressure and velocity distribution to minimize the risk of coronary insufficiency. The development of novel prostheses is one of the most promising goals – the optimisation of surgical technique of coronary reinsertion another one. Our results show that variation of the outlet angle of the coronary artery fails to result in any significant enhancement of pressure or velocity distributions. Furthermore, it seems doubtful that the sinus design has any relevant advantage to the currently widely used "straight tube" model. This leads us to the following conclusion: It is probably not necessary to create new forms of artificial root prosthesis. Rather, it would seem primarily essential to search for elastic materials of similar characteristics as the native aorta.

The model geometries we used for the fluid dynamics simulations idealise the real anatomy and physiology, but they are in part better comparable with the artificial prostheses. This means, especially: (1) The walls of the models are rigid. (2) Blood is treaten as non-compressible fluid, so that density is constant and the easy-to-apply massflow continuous equation can be used. (3) Blood pressure is assumed to be homogeneous as it occurs in a lying person at normal atmospherical pressure. (4) The viscosity of the blood depends on many factors, like temperature, velocity and haemo-composition. In this model it is assumed to be an ideal Newtonian fluid that does not depend on temperature and volume flow. The radial component of the law of Hagen-Poiseuille is therefore neglected. (5) The flow distribution at the inlet of the aortic valve region is idealised to a laminar flow. Turbulent components are calculated inside the model.

In real geometries, the walls of the models are not rigid, the blood pressure is not homogeneous and the flow is not a laminar flow. Nevertheless, these assumptions were made for this study because the effect should be similar in both models and should not lead to significant differences between the models. Therefore, we neglected these effects and made the assumptions. In future studies these effects could be included in more sophisticated simulations to determine even more realistic values, although generally the tendency of the results will not be significantly influenced.

## Conclusion

Our studies suggest that the variation of the coronary outlet angles in prosthesis with the "Windkessel" sinus design does not lead to useful improved pressure and velocity distributions, neither in the standard tube nor in the tube with sinus design. Surgeons should feel free to vary the outlet angle within the anatomic structural conditions for the technique of coronary reinsertion.

## Abbreviations

CFD: Computational fluid dynamics; M1: model 1; M2: model 2.

## Competing interests

The author(s) declare that they have no competing interests.

## Authors' contributions

JFV completed the technical part of this study, implementing and analyzing the fluid dynamic models. CB initialized the basic idea and did the medical part. Both authors read and approved the final manuscript.
